# Enhancing probiotic impact: engineering *Saccharomyces boulardii* for optimal acetic acid production and gastric passage tolerance

**DOI:** 10.1128/aem.00325-24

**Published:** 2024-05-16

**Authors:** Bruna Trindade de Carvalho, Ana Subotić, Paul Vandecruys, Sara Deleu, Séverine Vermeire, Johan M. Thevelein

**Affiliations:** 1 NovelYeast bv, Bio-Incubator BIO4, Leuven-Heverlee, Belgium; 2 Laboratory of Molecular Cell Biology, Institute of Botany and Microbiology, KU Leuven, Leuven-Heverlee, Belgium; 3 Department of Chronic Diseases, Metabolism & Ageing (CHROMETA), KU Leuven, Leuven, Belgium; 4 Center for Microbiology, VIB, Leuven-Heverlee, Belgium; Chalmers tekniska hogskola AB, Gothenburg, Sweden

**Keywords:** *Saccharomyces boulardii*, probiotics, acetic acid, acid tolerance

## Abstract

*Saccharomyces boulardii* has been a subject of growing interest due to its potential as a probiotic microorganism with applications in gastrointestinal health, but the molecular cause for its probiotic potency has remained elusive. The recent discovery that *S. boulardii* contains unique mutations causing high acetic acid accumulation and inhibition of bacterial growth provides a possible clue. The natural *S. boulardii* isolates Sb.P and Sb.A are homozygous for the recessive mutation *whi2*^S270*^ and accumulate unusually high amounts of acetic acid, which strongly inhibit bacterial growth. However, the homozygous *whi2*^S270*^ mutation also leads to acetic acid sensitivity and acid sensitivity in general. In the present study, we have constructed a new *S. boulardii* strain, derived from the widely therapeutically used CMCN I-745 strain (isolated from the pharmaceutical product Enterol), producing even higher levels of acetic acid while keeping the same tolerance toward low pH as the parent Enterol (ENT) strain. This newly engineered strain, named ENT3, has a homozygous deletion of *ACH1* and strong overexpression of *ALD4*. It is also able to accumulate much higher acetic acid concentrations when growing on low glucose levels, in contrast to the ENT wild-type and Sb.P strains. Moreover, we show the antimicrobial capacity of ENT3 against gut pathogens *in vitro* and observed that higher acetic acid production might correlate with better persistence in the gut in healthy mice. These findings underscore the possible role of the unique acetic acid production and its potential for improvement of the probiotic action of *S. boulardii*.

**IMPORTANCE:**

Superior variants of the probiotic yeast *Saccharomyces boulardii* produce high levels of acetic acid, which inhibit the growth of bacterial pathogens. However, these strains also show increased acid sensitivity, which can compromise the viability of the cells during their passage through the stomach. In this work, we have developed by genetic engineering a variant of *Saccharomyces boulardii* that produces even higher levels of acetic acid and does not show enhanced acid sensitivity. We also show that the *S. boulardii* yeasts with higher acetic acid production persist longer in the gut, in agreement with a previous work indicating competition between probiotic yeast and bacteria for residence in the gut.

## INTRODUCTION

The human microbiome is a highly intricate ecosystem, comprised of thousands of different species and displaying considerable interindividual variation (1, 2). Maintaining a healthy microbiome is essential for overall well-being, and disturbances caused by factors such as antibiotic treatment have been linked to an increased risk of various diseases, including diabetes (3), Crohn’s disease (4), obesity (5) and allergies such as asthma (6–8).

Probiotics have long been utilized to modulate the microbiome and assist in recovery in particular from intestinal diseases like traveler’s diarrhea. The most commonly used probiotics include lactic acid bacteria, such as *Lactobacillus* spp. and *Bifidobacterium* spp., that are often ingested with fermented foods (9). Among eukaryotic microorganisms, *Saccharomyces cerevisiae* var. *boulardii* (*S. boulardii*) is the best known, studied, and commercialized probiotic species. An important advantage of the use of yeast as probiotic over bacterial probiotics is the yeast’s natural resistance to commonly used bacterial antibiotics, making it a preferred choice for patients suffering from antibiotic-induced diarrhea (10, 11). *S. boulardii* is commonly used in the treatment of pathogen-induced diarrhea (10, 12) and other gastro-intestinal (GI) disorders. Positive results have been observed in both Crohn’s disease (12) and ulcerative colitis (13, 14); however, strong efficacy data are lacking, and further optimization of probiotics is therefore required.

*S. boulardii* is believed to exert its beneficial effects on the gastrointestinal tract through various mechanisms, including direct action against pathogens, detoxification of pathogenic toxins, modulation of the gut microbiota, regulation of tight junction permeability, and activation of innate immunity (15). Two major potential mechanisms for the probiotic action were demonstrated: the binding to bacteria and toxins facilitating their elimination (16) and the secretion of specific proteins. These include a 54-kDa serine protease that cleaves toxins A and B of *Clostridium difficile*, a 63-kDa protein phosphatase that inactivates *E. coli* lipopolysaccharide (LPS), and a 120-kDa non-enzymatic protein that reduces cAMP formation in intestinal cells induced by cholera toxin (15, 17, 18). While the exact identity of the genes encoding these proteins remains to be elucidated, research suggests that they are not unique to *S. boulardii* but are also found in its close relative and non-probiotic species, *S. cerevisiae*, adding further enigma to the distinct probiotic effect of *S. boulardii* (19).

The main genomic differences between *S. boulardii* and *S. cerevisiae* are the absence of two hexose transporters and two maltose-, three palatinose-, and four asparagine-utilization genes (20, 21). However, the absence of these genes does not provide any obvious link with possible probiotic action. An interesting divergence is the presence of a point mutation in the *PGM2* gene in all sequenced *S. boulardii* strains, which is responsible for its defective growth on galactose, but also provides superior temperature tolerance and faster growth at 37°C compared with *S. cerevisiae* (22). The ability to thrive at human body temperature is a crucial requirement for probiotic microorganisms.

A recent breakthrough in our understanding of *S. boulardii’s* unique properties is the discovery of its high acetic acid production, a characteristic absent in *S. cerevisiae* (23). This phenotype relies on two mutations, *sdh1^F317Y^* and whi2^S287*^, present in two copies in all and one copy in most *S. boulardii* strains, respectively. Two natural isolates, Sb.P and Sb.A, harbor two copies of the whi2^S287*^ allele and produce unusually high levels of acetic acid, a compound known to enhance intestinal epithelial defense and thus protect against infections (24, 25). Acetate is also a crucial substrate in the gut for the production of butyrate, a short-chain fatty acid (SCFA) with known anti-inflammatory properties, as it promotes regulatory T cell function (26). Additionally, high acetic acid production may contribute to the reduction of pH in the GI lumen, a well-recognized mechanism of action against pathogens like *Salmonella*, *Vibrio cholerae*, and *Blastocystis* (20). Accordingly, high acetate concentrations have shown anti-inflammatory and barrier-protective effects on organoid-derived epithelial monolayer cultures from patients with ulcerative colitis (27). On the other hand, the homozygous whi2^S287*^ allele in the Sb.P and Sb.A strains of *S. boulardii* also causes higher sensitivity to acetic acid and acid in general (23), possibly compromising their survival during passage through the stomach.

In the present work, we therefore aimed to construct by targeted genetic engineering an alternative *S. boulardii* variant from the widely therapeutically used CMCN I-745 strain (pharmaceutical product Enterol) that accumulates even higher acetic acid concentrations while keeping similar acid tolerance as the commercial parent strain. We show that the newly engineered strain has also higher antibacterial activity *in vitro* and better persistence *in vivo* in mice, which underscores the importance of acetic acid production for the probiotic action of *S. boulardii*.

## RESULTS

### Higher acetic acid production in *S. boulardii* strains correlates with enhanced persistence in mice intestine

**Fig 1:**
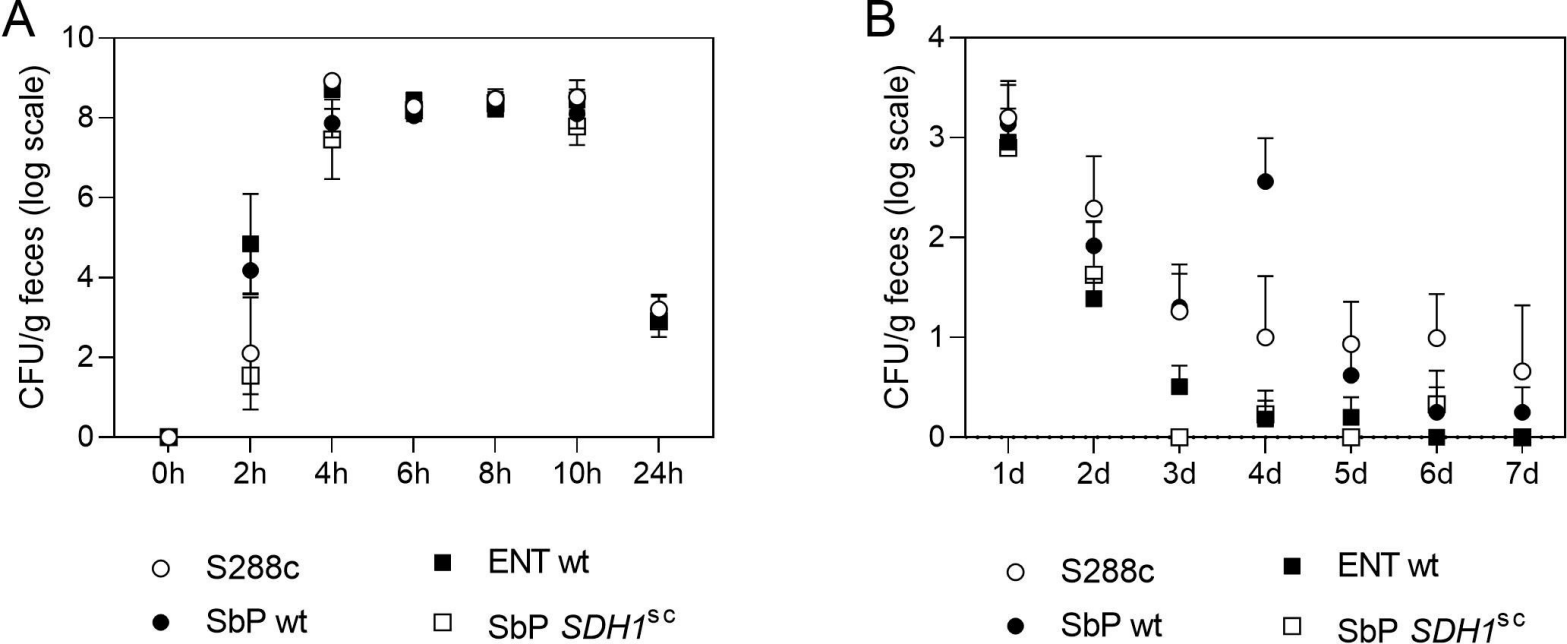
Concentration of live yeast cells recovered from mice feces following single-dose administration. (**A**) Feces collected during the first 24 h to investigate yeast strain survival. (**B**) Feces collected once a day during 1 week to investigate strain persistence in the gut.

We evaluated the survival (Fig. 1A) and persistence (Fig. 1B) through the mouse GI tract of the *S. boulardii* strains: Sb.P (high acetic acid), ENT (intermediate acetic acid), and Sb.P *SDH1^sc^* (no acetic acid) and the *S. cerevisiae* strain S288c. For that purpose, healthy mice received a single dose of yeast suspension via gavage, with a total of 10^9^ CFU/mouse.

Short-term survival of *S. cerevisiae* S288c (and also SbP *SDH1*^sc^) in feces collected after 2 h, likely reflecting acid tolerance during passage in the stomach, was more than 50% lower than that of SbP wt and ENT wt, which fits with literature data reporting higher acid tolerance of *S. boulardii* compared with *S. cerevisiae* (28–31).

However, fecal levels became similar for all tested strains, ranging around 10^8^ CFU/g feces in samples collected at 6, 8, and 10 h, followed by a drop at 24 h post-administration to around 10^3^ CFU/g feces. *S. cerevisiae* S288c and *S. boulardii* Sb.P were the only strains recovered on days 6 and 7 and were consistently found in higher amounts from day 2 onwards, indicating a better persistence and adhesion to epithelium. S288c showed the highest overall persistence while among *S. boulardii* strains, we observed a positive correlation between acetic acid production capacity and gut persistence (Fig. 1B).

### Acid sensitivity of SbP prevented further enhancement of its acetic acid production

**Fig 2:**
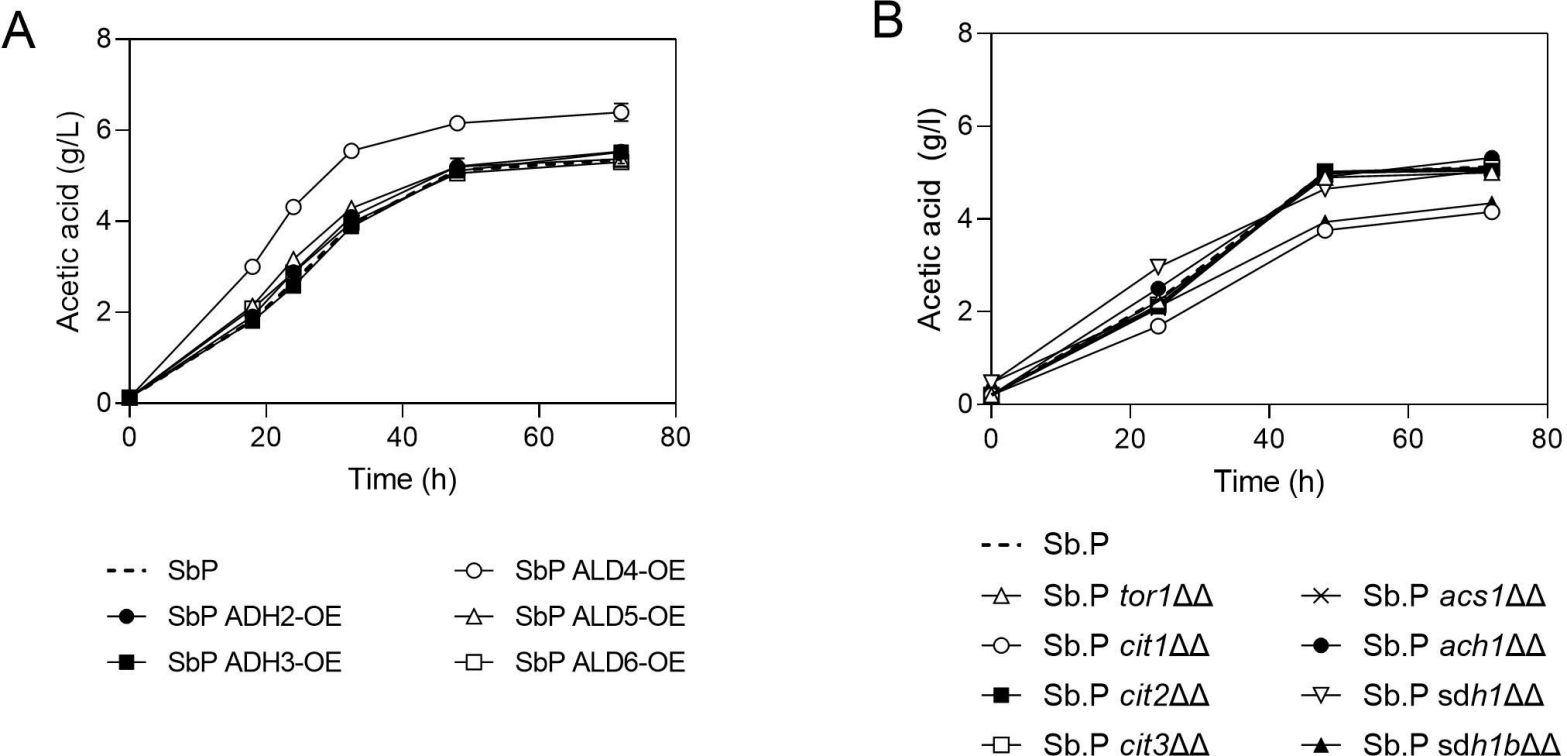
Evaluation of acetic acid accumulation in the Sb.P background by modification of selected targets. (**A**) Overexpression and (**B**) deletion. Cells were propagated in YPD2% at 37°C, 200 rpm for 72 h. Results are the mean of three biological replicates.

To boost acetic acid production in Sb.P, we explored the overexpression and deletion of 13 candidate genes, being 5 targets whose overexpression might shift the glycolytic metabolism toward more acetate production (*ADH2*, *ADH3*, *ALD4*, *ALD5*, and *ALD6*) and 8 targets whose deletion might do the same (*CIT1*, *CIT2*, *CIT3*, *ACH1*, *SDH1*, *SDH1b*, *ACS1*, and *TOR1*). Among all tested modifications, only the overexpression of the *ALD4* gene proved effective in increasing acetic acid accumulation in Sb.P, as illustrated in Fig. 2A. *SDH1b* and *CIT1* deletion resulted in decreased acetic acid accumulation; the latter also negatively affected propagation. All the other modifications did not affect acetic acid accumulation or cell growth (Fig. 2B). However, the previously reported acid sensitivity of Sb.P (23) raises concerns about the feasibility of further increasing acetic acid production in this strain without compromising its viability.

### Impairing TCA activity enhances acetate accumulation in *S. boulardii*

**Fig 3:**
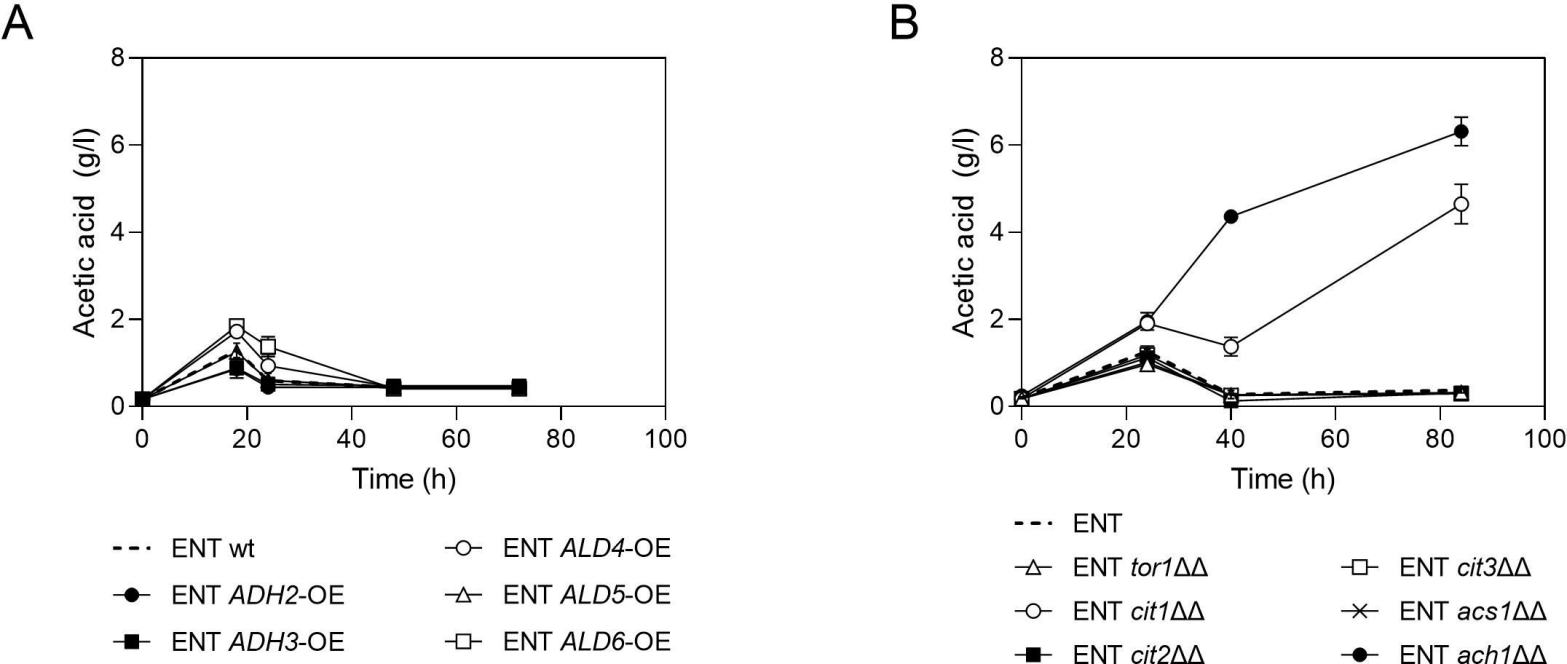
Evaluation of acetic acid accumulation in the ENT background caused by modification of selected targets. (**A**) overexpression and (**B**) deletion. Cells were propagated in YPD2% at 37°C, 200 rpm for 72 h. Results are the mean of three biological replicates (the ENT ach1ΔΔ strain used in panel B is colony 2; see Fig. S1).

In an effort to engineer a strain with an increased capacity for acetate production while avoiding the acid sensitivity observed in Sb.P, we applied the same genetic modifications to the ENT background. Among the overexpression targets, *ALD4* and *ALD6* exhibited only marginal increases in acetate accumulation (Fig. 3A), and this increase was also transient. Interestingly, when the *CIT1* or *ACH1* gene was deleted, a dramatic increase in acetate production was observed, and the cells also switched from transient to permanent acetate accumulation within the time frame of the experiment in yeast extract peptone dextrose (YPD)2% (Fig. 3B).

Notably, *CIT1* deletion led to lower and slower acetate accumulation in the Sb.P background (Fig. 2B), but in the ENT background, it resulted in a transition from low and transient to high and sustained accumulation, albeit at a slower rate (Fig. 3B). As observed in Sb.P, *CIT1* deletion in the ENT background also resulted in a partial growth defect. Following 48 h of propagation in YPD2% at 30°C, the ENT *cit1ΔΔ* achieved approximately half of the cell density of the ENT wild type. Specifically, while the ENT wild type reached an optical density at 600 nm (OD_600_) of 39, the ENT cit1ΔΔ strain only attained an OD_600_ of 21.

On the other hand, *ACH1* deletion did not interfere with propagation capacity. Nonetheless, acetic acid accumulation resulting from *ach1∆∆* was found to be a highly variable phenotype, with remarkable deviations between independent transformants (Fig. S1A). Some of these transformants also displayed variable acetate accumulation when tested in replicate experiments (Fig. S1B). One top-performing and stable transformant (colony 2), designated ENT1, was selected for further improvement. ENT1 already accumulated higher levels of acetate compared with Sb.P. Interestingly, *ACH1* deletion in Sb.P resulted in a stable phenotype, with all transformants showing a similar acetate accumulation pattern as displayed by the wild-type Sb.P strain (Fig. S2).

### *ALD4* overexpression enhances acetate accumulation when acetate utilization is impaired

Among the tested modifications, the overexpression of the *ALD4* gene was the sole alteration that further boosted acetate production in Sb.P. However, when *ALD4* was overexpressed in the ENT background, the increase in acetate accumulation was only slightly higher and remained temporary (Fig. 2 and 3).

Nevertheless, *ALD4* overexpression proved effective in enhancing acetate accumulation in the ENT background when TCA cycle activity was compromised by *ACH1* deletion. The *ALD4* gene was expressed under the strong and constitutive *TEF1* promoter, with two copies of the *ALD4*-OE construct integrated into the ENT1 strain (ENT ach1ΔΔ), yielding the ENT2 strain. Subsequently, two more copies were integrated in ENT2, yielding the strain ENT3.

**Fig 4:**
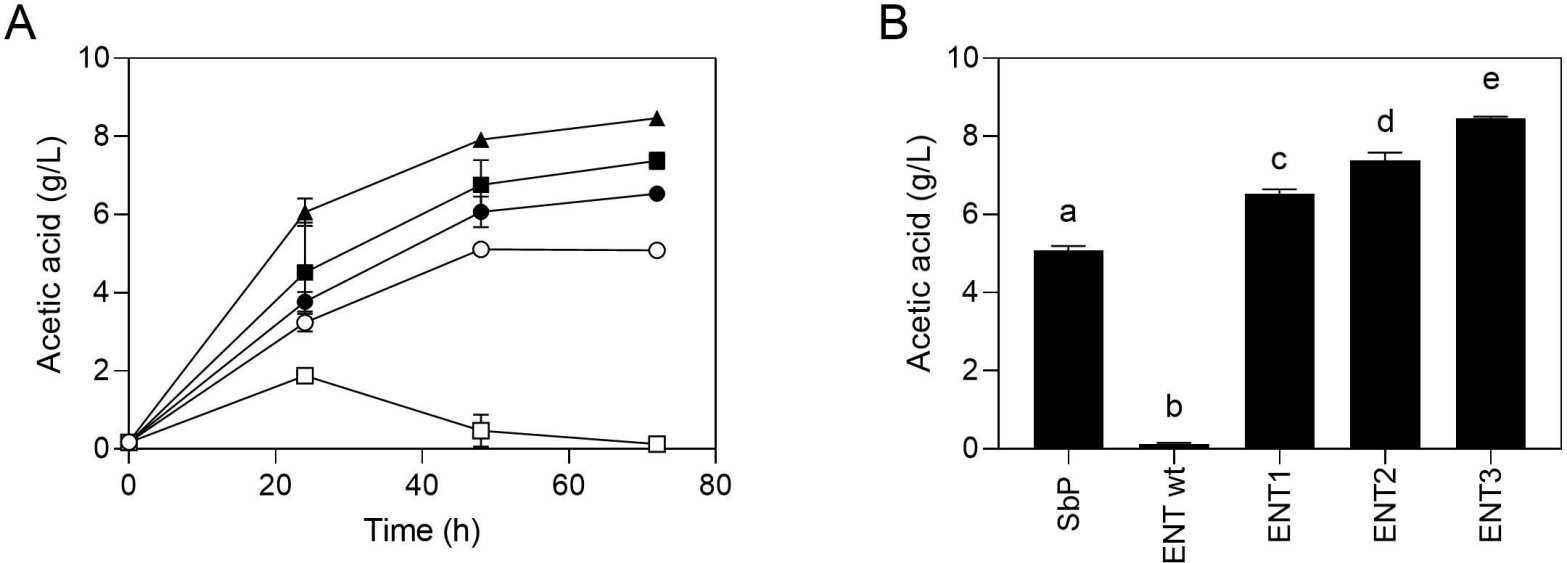
Effect of ALD4 overexpression on the ENT1 strain on acetate accumulation. (**A**) Acetate accumulation as a function of time. □, ENT wild type; ○, Sb.P; ●, ENT1; ■, ENT2; ▲, ENT3. (**B**) Acetate accumulation at 72 h. Cells were propagated in YPD2% at 37°C, 200 rpm for 72 h. Results are the mean of three biological replicates. Different letters indicate a significant difference between groups (*P* value < 0.001 for all groups compared with each other using one-way ANOVA, followed by Tukey’s multiple comparisons test).

The impact of *ALD4* overexpression was dose dependent. Presence of two copies of the ALD4-OE construct in strain ENT2 resulted in a 14% increase in acetate production compared with the precursor strain, ENT1. Subsequent integration of two more copies, forming strain ENT3, led to a remarkable 30% boost in acetate production compared with ENT1 and a substantial 70% increase compared with the Sb.P strain (Fig. 4A and B).

This dose-dependent effect was also observed at the expression level (Fig. S3). Higher *ALD4* expression was observed in ENT2 and ENT3 at 8 h of growth, with a notable discrepancy between ENT2 and ENT3 becoming more pronounced at 24 h. The enhanced expression of *ALD4* correlated with increased acetate production, further influencing a lower fermentation pH. Specifically, the pH of the supernatant for ENT, ENT1, ENT2, and ENT3 was 4.69, 4.08, 3.93, and 3.73, respectively.

### ENT3 is the only strain capable of permanent acetate accumulation in low glucose concentrations

**Fig 5:**
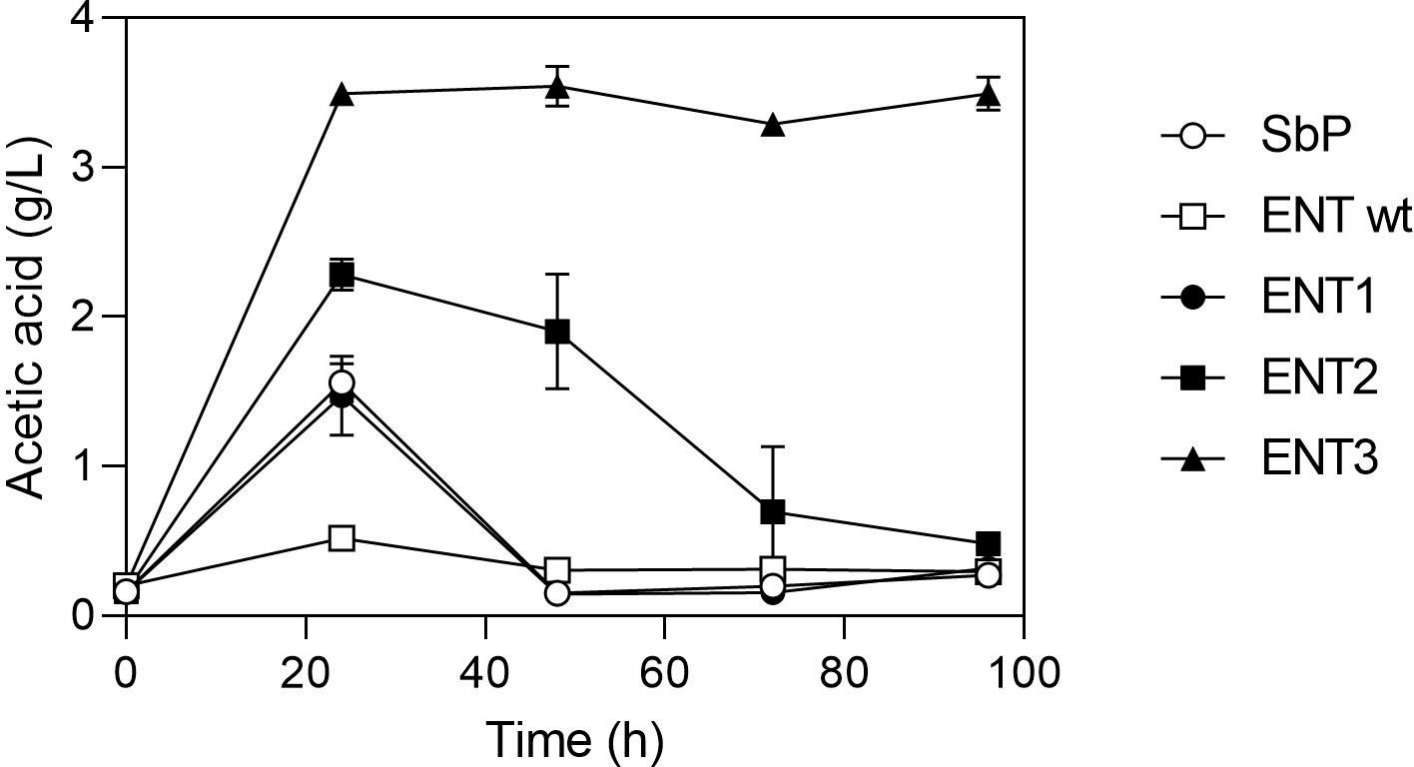
Effect of ALD4 overexpression on acetate accumulation in medium with a low glucose concentration. Cells were propagated in YPD0.9% (50mM) at 37°C, 200 rpm for 72 h. Results are the mean of three biological replicates.

Under cultivation in YPD0.9% (50mM glucose), it was observed that Sb.P could only transiently accumulate acetate, while the ENT wild type did not accumulate significant levels. Upon deletion of the *ACH1* gene (ENT1), the strain exhibited a profile similar to that of Sb.P, with maximal acetate production reaching approximately 1.5 g/L at 24 h. Substantially higher but still transient accumulation was observed in ENT2, whereas the ENT3 strain demonstrated the ability to persistently accumulate approximately 3.5 g/L of acetate (Fig. 5).

**Fig 6:**
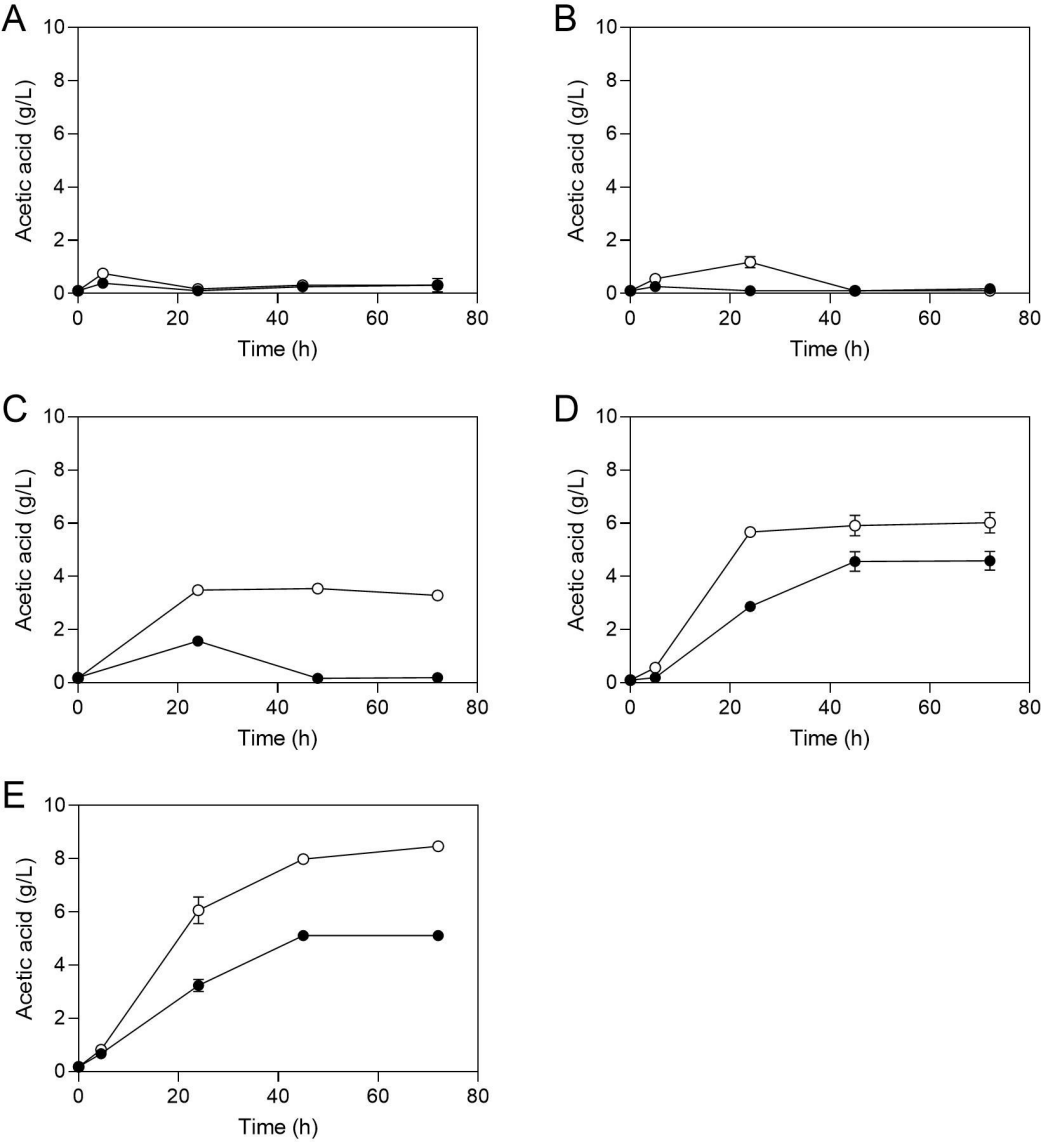
Comparison of Sb.P (●) and ENT3 (○) performance for acetate accumulation at different glucose levels. (**A**) 5 mM, (**B**) 20 mM, (**C**) 50 mM, (**D**) 75 mM, and (**E**) 110 mM. Cells were propagated in YP with different glucose levels at 37°C, 200 rpm for 72 h. Results are the mean of two biological replicates.

Furthermore, a comparison of Sb.P and ENT3 at various glucose concentrations revealed that ENT3 consistently outperformed Sb.P in acetate accumulation (Fig. 6). At the lowest tested glucose concentration (5 mM), none of the strains was able to accumulate acetate (panel A). At 20 mM (0.36%), only ENT3 could transiently accumulate acetate (panel B), and at 50 mM (0.9%), ENT3 was able to permanently accumulate acetate, whereas Sb.P exhibited lower and still transient accumulation (panel C). Beyond 75 mM (1.35%), both strains could permanently accumulate acetate, but ENT3 consistently demonstrated superior accumulation (panel D), with the disparity between the two strains becoming more pronounced at 110 mM of sugar (2%, panel E).

### ENT3 exhibits inhibitory capacity against gut-isolated pathobiont bacteria

**Fig 7:**
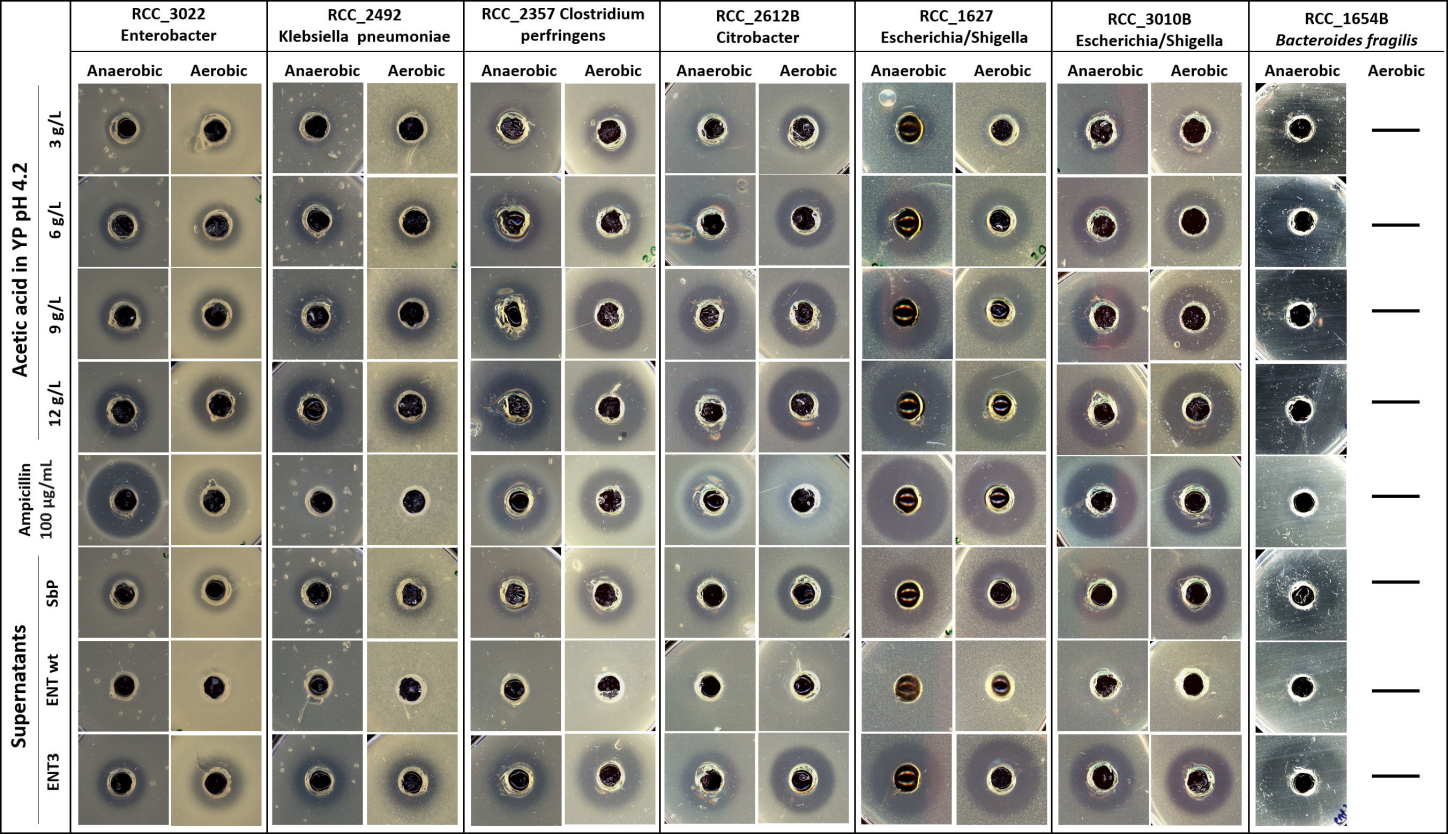
Agar well diffusion assay. Nutrient Schaedler agar was inoculated with each one of the seven pathogenic bacteria strains tested. Wells were punched into the agar and filled with 100 µg/mL ampicillin (control), pure acetic acid (3, 6, 9, and 12 g/L, in YP pH 4.2), or cell-free supernatant from Sb.P and ENT3. Two sets of plates were prepared, from which one was incubated inside an anaerobic jar. Plates were incubated at 37°C for 24–48 h.

We have previously shown that acetic acid at the concentration of 6 g/L, as well as Sb.P fermentation medium supernatant, could inhibit the growth of *Escherichia coli* MG1655 (23). We extended this investigation to explore the efficiency of acetic acid as an antimicrobial agent against potential pathobionts isolated from the gut. Different concentrations of acetic acid and Sb.P and ENT3 fermentation medium supernatants were tested with the agar-well diffusion assay in the presence of gut bacteria (Fig. 7).

For this test, appropriate agar medium (Schaedler) was inoculated with each one of the tested bacterial strains. Wells were punched into the agar and filled with 100 µg/mL ampicillin (control), acetic acid at 3, 6, 9, or 12 g/L in YP pH 4, or fermentation medium supernatant of the Sb.P and ENT3 strains. Two sets of plates were prepared, one of which was incubated inside an anaerobic jar. All plates were incubated at 37°C.

Acetic acid demonstrated effectiveness against all tested potential pathobionts. As expected, higher acetic acid concentrations yielded larger inhibition zones, indicating that the antimicrobial activity was attributed to acetic acid rather than the YP pH4 medium. All tested strains, with the exception of *Klebsiella pneumoniae*, were inhibited by ampicillin, and the strains exhibited higher sensitivity to antibiotics in the absence of oxygen, resulting in larger inhibition zones (Fig. 7). Acetic acid efficacy, however, was slightly affected, gaining or losing potency depending on the strain.

The Sb.P and ENT3 supernatants were also effective against all tested strains, with ENT3 consistently displaying stronger antimicrobial potency compared with Sb.P. *Klebsiella pneumoniae* exhibited resistance to ampicillin but sensitivity to high levels of acetic acid, including ENT3 supernatant, and this sensitivity was unaffected by oxygen.

### Engineering of ENT for high-acetic acid production without compromising cell viability at low pH

An ideal probiotic microorganism should be tolerant to the low pH of gastric fluid in order to survive gastric passage as some probiotic actions depend on cell viability or metabolic activity of the yeast, such as secretion of antitoxin proteins and antimicrobial agents like acetic acid.

To assess whether engineering ENT for high acetic acid accumulation had compromised its tolerance to low pH, we simulated gastric passage by exposing strains to a saline solution (NaCl 0.5%) at pH 1.7 for 3 h at 37°C. Viability was determined by colony counting on nutrient agar plates of cells that had been exposed for 3 h to acidic saline (NaCl 0.5%, pH1.7) compared with 3 h exposure to saline at pH 6.8. In this way, only the effect of low pH is taken into consideration.

**Fig 8:**
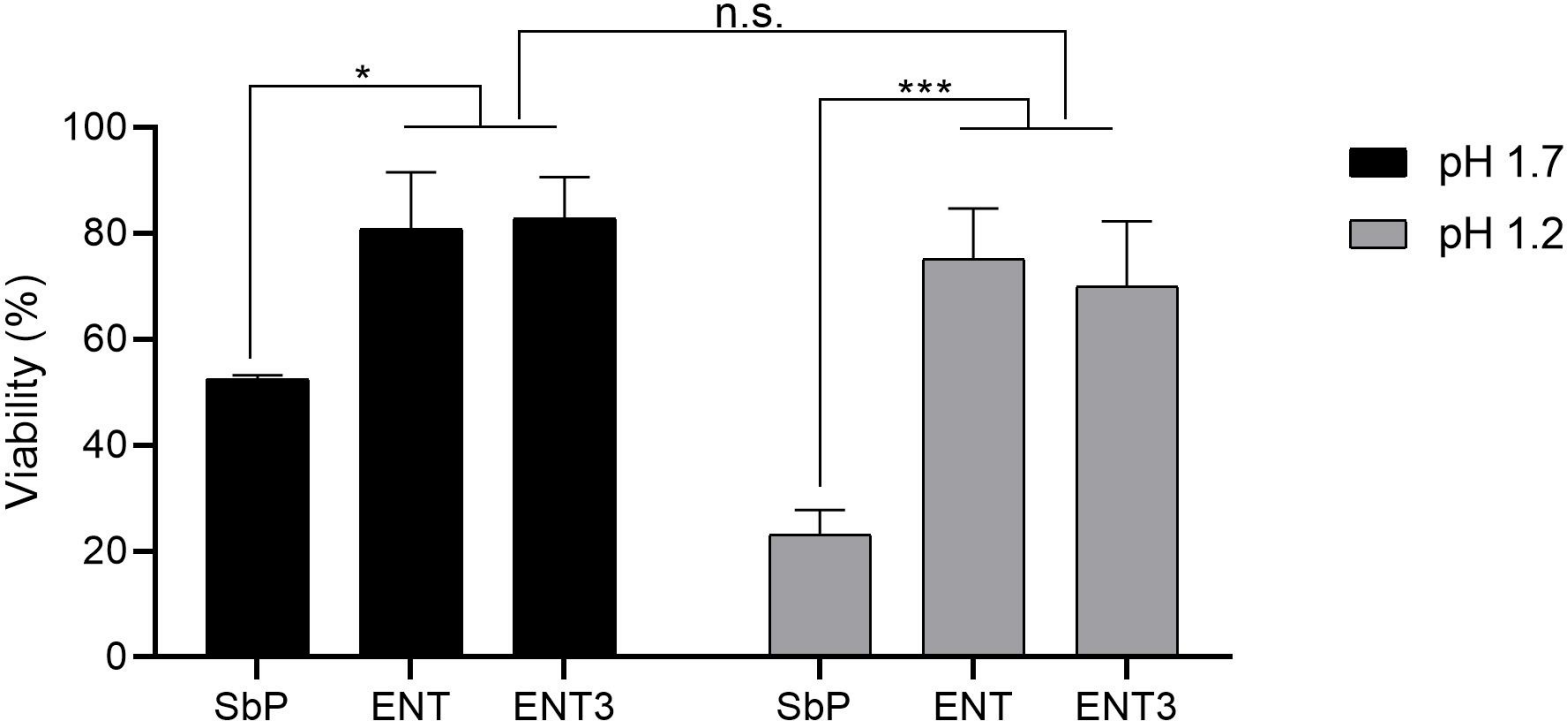
Viability after exposure to simulated gastric acid. Tolerance to the low pH of simulated gastric acid was determined in saline (NaCl 0.5%) at pH 1.7 and pH 1.2. Results were obtained by comparing CFU of cells exposed to neutral saline (NaCl 0.5%, pH 6.8) to the CFU of cells exposed to acidic saline. Cells were incubated for 3 h at 37°C. All groups compared with each other using two-way ANOVA with Sidak’s multiple-comparison test (*P ≤ 0.05 and ***P ≤ 0.001; ns, non-significant).

Surprisingly, S288c showed nearly unaffected viability. The engineering of ENT3 for high acetate production did not compromise its resistance to low pH, with the ENT wild type and ENT3 exhibiting about 80% viability, while Sb.P and Sb.P *SDH1^sc^* showed about 50% viability (Fig. 8 and data not shown).

### ENT3 modifications have the same effect in other strain backgrounds

**Fig 9:**
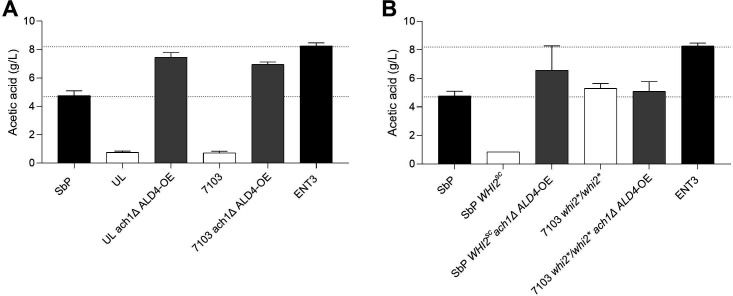
Effect of ENT3 modifications on other *S. boulardii* strains. (A) Effect of *ach1Δ ALD4-*OE (4x) on wild-type *S. boulardii* strains. (B) Effect of the whi2* allele on the acetate production phenotype generated by the ENT3 modifications. Cells were propagated in YPD2% at 37°C, 200 rpm for 72 h.

We demonstrated that the combination of *ACH1* gene deletion with the integration of TEF1p-ALD4-CYC1t cassette four times in the genome was responsible for the very high acetic acid accumulation in the Enterol background, resulting in the strain called ENT3. To assess the universality of the high acetate phenotype generated in ENT3, we introduced the same modifications into two other wild-type *S. boulardii* strains (WHI2/whi2*), UL and 7103 (Fig. 9). In both backgrounds, UL and 7103, the engineered strains also exhibited very high acetate accumulation, suggesting that these modifications are broadly applicable to generate high-acetate-producing strains (Fig. 9A).

Furthermore, we examined the impact of the whi2* allele on the phenotype generated by *ach1Δ ALD4-OE* (Fig. 9B). As previously mentioned, the homozygous whi2* allele is responsible for the high acetate phenotype shown by SbP, but it is also responsible for its susceptibility toward low pH. Therefore, we introduced these modifications into Sb.P, in which both whi2* alleles were replaced by the *S. cerevisiae* wild-type allele *WHI2*. This alteration allowed Sb.P to achieve high acetate accumulation, eliminating the whi2*-induced acid sensitivity. We also evaluated the ENT3 modifications in the 7103 whi2*/whi2* strain and found that acetic acid accumulation remained the same (Fig. 9B). This observation underscores the limiting effect of the homozygous whi2* allele on acetate accumulation levels.

## DISCUSSION

Survival and persistence within the gastrointestinal tract are pivotal factors influencing the effectiveness of probiotic strains. To address this, we investigated the survival of *S. boulardii* strains after passage through the mice GI tract and the possible influence of acetic acid on this trait. We evaluated the survival of Sb.P, characterized by high acetic acid production yet sensitivity to low pH; ENT, isolated from the pharmaceutical product Enterol, exhibiting intermediate acetic acid production and robustness to pH fluctuations; and Sb.P *SDH1^sc^*, a genetically modified strain of SbP lacking acetic acid production. Additionally, we included *S. cerevisiae* S288c in the experiment to serve as a control for adherence due to its well-studied nature and genetic similarity to *S. boulardii*, albeit lacking probiotic capabilities.

At 2 h post-gavage, a large variation in yeast counts was observed as a consequence of differences in transit time. Fecal yeast levels peaked from 4 to 10 h, reaching approximately 10^8^ CFU/g feces, subsequently declining to around 10^3^ CFU/g at 24 h. These findings align with reports in the literature showing complete clearance of the murine GI tract between 6 and 8 h (32).

All tested strains, regardless of their intrinsic sensitivity to low pH, demonstrated similar fecal levels between 6 and 24 h, indicating comparable survival during gastrointestinal passage. Notably, mice have a considerably higher gastric pH than humans, reaching approximately 3.0 in the fed state and 4.0 when fasted (33). Therefore, the observed differences in pH sensitivity, particularly in Sb.P, are likely to be less pronounced in the mouse model. It is interesting to note that fecal levels exceeding 5 × 10^6^ CFU/g have been associated with protective effects on the germ-free mouse model for pseudomembranous colitis (34).

*S. boulardii* and *S. cerevisiae* were reported as being unable to adhere to human and mouse epithelial cells in the presence of a normal microbiome (29, 35), and no viable yeast cells were found in the feces 48 h post-gavage in mice (29). Therefore, the low cell counts encountered after 24 h are not surprising. Nevertheless, differences between the strains could be observed. Both *S. boulardii* Sb.P and *S. cerevisiae* S288c were consistently found in higher amounts from day 2 onwards, indicating better persistence and adhesion to the epithelium. Intriguingly, Sb.P and Sb.P *SDH1^sc^*, which differ only in one SNP, displayed a strong difference in persistence, with Sb.P showing much higher persistence compared with Sb.P *SDH1^sc^*. This suggests that the acetic acid production of *S. boulardii* may facilitate its competition with bacteria for epithelial membrane adhesion.

Surprisingly, *S. cerevisiae* S288c showed the highest long-term persistence in the gut, in spite of lower survival in the short term in feces collected after 2 h. *S. cerevisiae* S288c has never been reported to possess probiotic potential. However, probiotic activity is certainly not determined solely by persistence in the gut. Probiotic activity is a complex trait of which better persistence in the gut may be one of the supportive factors. We also do not claim that the high acetic acid production that we discovered in *S. boulardii* (23) is the only factor determining its superior probiotic potential compared with *S. cerevisiae*, but it may likely be a major factor and act synergistically with persistence in the gut.

It is well known that acetic acid is more toxic at low pH. In order to reach the same toxicity at higher pH values, much higher concentrations of acetic acid are required (36, 37). The average pH of the gut is neutral to slightly alkaline, but as discussed for the issue of the aerobic versus anaerobic conditions (see below), we believe that *S. boulardii* adheres to the intestinal epithelium (like many other microbes) and is therefore present in the microenvironment between the epithelial invaginations and (micro)villi in the brush border, in which the pH may be lower compared with the bulk of the medium in the gut lumen due to proton extrusion by the adhering microbes. Moreover, the high secretion of acetic acid by *S. boulardii* attached inside the epithelial invaginations in the brush border may lower the pH in this microenvironment significantly and thus increase the toxicity of acetic acid. In this local environment *S. boulardii* competes with bacteria for adherence to the epithelium and persistence in the gut. While most wild-type *S. boulardii* strains typically exhibit a supernatant pH of around 4.89 when cultivated in YPD2%, SbP and ENT1 demonstrate pH levels around 4.08, ENT2 shows 3.93, and ENT3 shows 3.78 (Table S1). This indicates that *S. boulardii* with higher acetic acid secretion has a higher potential to acidify the surrounding medium.

In our agar well diffusion assay, pure acetic acid was added to unfermented yeast extract peptone (YP) media without an additional carbon source at concentrations of 3, 6, 9, or 12 g/L. To standardize the proportion of dissociated and undissociated forms of acetic acid, the pH of each medium was adjusted to 4.0. This pH selection was based on the average pH of supernatants from high acetic acid-producing strains. As expected, higher concentrations of acetic acid led to larger inhibition zones, indicating that the antimicrobial activity was primarily due to acetic acid rather than any other component of the YP pH4 medium that remained the same in the different test media. Additionally, the inhibitory effect of acetic acid was assessed independently of other potentially antimicrobial compounds produced by the yeast, as unfermented YP served as the vehicle. The antimicrobial effect of ethanol was not evaluated, as only 72-h fermentation supernatant was utilized in the diffusion assay, and by this time, ethanol had been consumed by all the yeasts (Fig. S4). No other fermentation compounds appeared to have antimicrobial effects, as evidenced by the absence of inhibition zones in the ENT supernatant. Similarly, Offei et al. (23) also reported no inhibition by the supernatant of *S. cerevisiae* and low-acetic acid-producing *S. boulardii* strains.

In the WHI2/whi2* S. boulardii strains, the acetic acid accumulation is only transient while in the whi2*/whi2* S. boulardii strains, the acetic acid accumulation is persistent. The transient character is due to consumption of the acetic acid, while the whi2*/whi2* S. boulardii strains are unable to consume acetic acid at 37°C, unlike the other *S. boulardii* strains and unlike many *S. cerevisiae* strains that are capable of growth on acetate at both 30°C and 37°C (23). The new strains with high acetic acid accumulation that we now constructed all showed persistent acetic acid accumulation in medium with high glucose. In medium with a low glucose level, weak and transient accumulation of acetic acid was observed under some conditions for the ENT2 and ENT3 strains, but also for the Sb.P strain, indicating that the three strains are able to consume low levels of acetic acid (Fig. 5 and 6). Possibly, the strong acidification caused by the high levels of acetic acid produced in media with high glucose might also play a role in the persistent character of the acetic acid accumulation by compromising the reutilization of the acetic acid.

We have attempted to increase acetic acid production in *S. boulardii* strains by overexpression of candidate genes (*ADH2*, *ADH3*, *ALD4*, *ALD5*, and *ALD6*) or deletion of candidate genes (*CIT1*, *CIT2*, *CIT3*, *ACH1*, *SDH1*, *SDH1b*, *ACS1*, and *TOR1*) all involved in acetic acid production or consumption pathways in yeast metabolism (Fig. S5). Several genes belong to the TCA cycle, and the role of the TCA cycle in the probiotic capacity of *S. boulardii* was further investigated. The deletion of *CIT1*, responsible for catalyzing the initial TCA cycle step by combining acetyl coenzyme A and oxaloacetate to form citrate, resulted in elevated acetate levels but exhibited a slow accumulation pattern and compromised propagation. Conversely, the disruption of the TCA cycle through the deletion of *ACH1* yielded a distinct response. *ACH1* encodes a protein with CoA transferase activity, required for acetate utilization and, when impaired, prompted the redirection of acetyl-CoA toward acetic acid production at a higher rate and without hindering propagation.

Our results also demonstrate that *ALD4* overexpression in Sb.P was effective in enhancing acetate accumulation, even when the TCA cycle activity was not impaired. *ALD4*, as a mitochondrial aldehyde dehydrogenase required for growth on ethanol, plays a crucial role in acetate metabolism. It catalyzes the conversion of acetaldehyde to acetate, and its expression is repressed by glucose. *ALD4* overexpression in ENT, however, could only cause a slight and still transient increase. Notably, when acetate utilization in the TCA cycle was impaired by *ACH1* deletion, *ALD4* overexpression led to higher acetate accumulation in a dose-dependent way. These results suggest that targeted genetic modifications can be leveraged to optimize acetate production in *S. boulardii* strains, providing a basis for further engineering efforts.

In the context of acetic acid production enhancement, the whi2^*^ allele emerged as a critical factor. This mutation not only compromised growth but also rendered Sb.P highly sensitive to acetic acid, resulting in a mere 20% cell viability at the end of fermentation at 37°C. In contrast, other *S. boulardii* strains, including ENT, which are heterozygous for WHI2/whi2*, and the *S. cerevisiae* strain ER (*WHI2/WHI2*), exhibited nearly 100% cell viability (23). This acid sensitivity also prevented further acetic acid accumulation, not only in SbP but also in the 7103 strain.

According to our hypothesis, a key aspect of the probiotic capacity of *S. boulardii* relies on the capacity to produce acetic acid from glucose. The concentration of glucose in the intestinal lumen is a dynamic parameter that varies according to physiological conditions and food intake. Luminal glucose concentrations were reported to range from 0.2 to 48 mM (0.036%–0.86% wt/vol) under all physiological conditions and claimed not to exceed 100 mM (1.8% wt/vol) even under the most unphysiological condition examined (38). However, Ferraris et al. (38) measured the overall luminal glucose concentration by simply emptying the gut content, while the glucose concentration in the unstirred layer of the brush border may be much higher because all major carbohydrases are attached to the epithelial cell membrane (39). Direct measurement of these concentrations is challenging due to the rapid uptake of glucose through the epithelium and its diffusion within the gut, but indirect measurements suggest that local glucose concentrations could reach levels of up to 300 mM (5.4%) (40). Our previous research demonstrated that higher sugar levels (4%) led to significant acetic acid accumulation in all *S. boulardii* strains, but not in most *S. cerevisiae* strains (23). This observation implies that *S. boulardii* strains can effectively accumulate acetic acid when glucose availability is high, for example, after a meal. However, our investigation has expanded to examine acetate accumulation under low glucose concentrations, such as those encountered in the brush border during the fasted state between meals. This analysis is significant as it suggests that ENT3 may exert a more prolonged probiotic effect by producing acetic acid even when glucose levels are low, a common occurrence during the fasting period.

Another critical aspect of probiotics is the ability to survive the harsh conditions of gastric passage, which includes demonstrating their robust resistance to low pH levels. The elimination of acetic acid production in Sb.P (SbP SDH1^*^) did not improve its low pH survival. The pH sensitivity displayed by Sb.P therefore appears to be due mainly to the *whi2*^S270*^ allele as the replacement of this allele with the *S. cerevisiae* wild-type allele *WHI2* successfully established tolerance to low pH. The *whi2*^S270*^ allele encodes a truncated protein, leading to acid sensitivity, a trait that aligns with previous findings where the *whi2*Δ strain exhibited substantially higher sensitivity to acetic acid than the wild type (23, 41).

ENT and its engineered strain ENT3, being heterozygous for WHI2/whi2*, do not exhibit the same high acid sensitivity as the Sb.P strain, which harbors two copies of the mutated allele whi2*. In our study, we tested the strains at pH 1.7, as this value was reported to be the median gastric pH in humans in the fasted state, with an interquartile range from 1.4 to 2.1 (42). The high ENT survival rate at low pH is also in accordance with results reported by Fietto et al. (28) demonstrating that the *S. boulardii* CNCM I-745 strain, the same genetic background as ENT, exhibits superior tolerance (75% viability) to low pH compared with the laboratory *S. cerevisiae* strain W303 (30% viability) in simulated gastric juice at pH 2 (28).

Remarkably, ENT3 also exhibits the capacity to accumulate acetic acid when grown under low glucose conditions, similar to the levels encountered in the post-meal fasting state. Furthermore, we have corroborated the antimicrobial potential of acetic acid against bacterial pathogens isolated from the gut. High acetic acid concentrations as well as the supernatant produced by SbP and ENT3 exhibited strong inhibitory capacity, even against *Klebsiella pneumoniae*, which was resistant to ampicillin. Similar results have recently been described by Chang et al. (43) for testing the antimicrobial effects of high SCFA concentrations *in vitro*. ENT3, which produces a higher concentration of acetic acid and lower pH, possesses a higher inhibitory capacity compared with Sb.P against all tested strains. The antimicrobial activity was not affected by the presence of oxygen, which is relevant to conditions in dysbiosis where the gut’s epithelial barrier is compromised, making the high-acetic acid-producing strains promising candidates for gastrointestinal health interventions. It is also worth to mention that the genetic modifications in the ENT3 strain that led to high acetic acid accumulation were successfully applied to other *S. boulardii* strains (UL and 7103), indicating that these modifications are transferable, and can potentially be used to construct novel probiotic strains with similar beneficial traits.

Some of the genes that we have modified encode enzymes of the TCA cycle that is only active under aerobic conditions. In our experiments, acetate production was measured under aerobic conditions. We have shown previously that the high acetic acid production that we discovered in *S. boulardii* is prevented by anaerobic conditions and that oxygen availability thus plays a critical role (23). Although the gut is often considered as a purely anaerobic environment, non-invasive measurements of the oxygen level have shown a radial distribution with high oxygen levels at the epithelial surface in the brush border followed by a steep decline in oxygen levels when moving to the center of the gut, rapidly reaching anaerobic conditions toward the center (44). This gradient is likely due to oxygen diffusing from the blood capillaries through the epithelial cells into the peripheral layer of the gut. *S. boulardii* cells that adhere to the epithelium in the brush border would therefore reside in an aerobic environment with a similar oxygen level as present in the epithelial cells and thus not be prevented of accumulating high acetic acid levels. Moreover, several reports have demonstrated increased oxygen availability in the gut under conditions compromising the presence of a healthy microbiome (45–47). Hence, under conditions in which *S. boulardii* is used as a probiotic, higher oxygen levels are likely present in the gut.

### Conclusions

Our investigation suggests that the newly identified set of modifications that confer high acetic acid accumulation without compromising acid tolerance holds potential for the development of superior probiotic strains. Our engineered ENT3 strain has demonstrated the ability to accumulate substantially higher levels of acetic acid compared with other *S. boulardii* strains. Additionally, ENT3 is capable of persistent acetic acid production under low glucose conditions, offering prospects for extended probiotic action during fasting intervals. Moreover, our research suggests a positive correlation between acetic acid production capability and the strain’s persistence in the gut. This raises the possibility that the high-acetic acid producer ENT3 may endure longer in the gastrointestinal tract, potentially extending its probiotic effects.

These collective findings not only enhance our understanding of the mechanisms underlying the probiotic activity of *S. boulardii* but also for the first time pave the way for targeted development of more effective probiotic strains with potential therapeutic application. The prospects for ENT3, with its unique acetic acid production traits, hold promise for the future improvement of probiotics as a therapeutic tool for gastrointestinal health.

## MATERIALS AND METHODS

Tables 1 to 3 list bacterial strains, yeast strains, and plasmids, respectively, used in this study. A list of gRNA target sequences and a list of donor DNAs are provided as Tables S2 and S3, respectively.

**TABLE 1:** Bacterial strains

RCC_no	Phylum	Family	Genus	Species
3022	Proteobacteria	Enterobacteriaceae	*Enterobacter*	sp.
2492	Proteobacteria	Enterobacteriaceae	*Klebsiella*	*pneumoniae* group
2357	Firmicutes	Clostridiaceae	*Clostridium*	*perfringens*
2612B	Proteobacteria	Enterobacteriaceae	*Citrobacter*	sp. (possibly *freundii*/*braakii*)
1627	Proteobacteria	Enterobacteriaceae	*Escherichia/Shigella*	
3010B	Proteobacteria	Enterobacteriaceae	*Escherichia/Shigella*	
1654B	Bacteroidetes	Bacteroidaceae	*Bacteroides*	*fragilis*

**TABLE 2:** Yeast strains^*a*^

Strain	Genotype	Description/phenotype	Origin/reference
Wild-type strains			
S288c	Wild-type *S. cerevisiae* strain	Widely used laboratory strain.	(48)
Sb.P	Wild-type *S. boulardii* strain	Natural isolate accumulating unusually high levels of acetic acid in YPD2%, sensitive to low pH.	Lene Jespersen, University of Copenhagen, Denmark
Enterol (ENT)	Wild-type *S. boulardii* strain	Commercial *S. boulardii*. Shows transient acetic acid production in YPD2%.	Isolated from Pharmacy product Enterol (23)
UL	Wild-type *S. boulardii* strain	(23, 31)	Niederberger, Nestlé (31)
7103	Wild-type *S. boulardii* strain	(23, 40)	Ultra-Levure batch 7103, Laboratoires Biocodex
Genetically modified strains			
Sb.P SDH1^Sc^	*SDH1*^Y202H, Y31F^/ *SDH1*^Y202H, Y31F^	Sb.P harboring two copies of the *SDH1* allele of S288c. Does not accumulate acetic acid.	(23)
SbP *ADH2*-OE	TEFp-ADH2-CYC1t::IS2.1	SbP strain harboring the *ADH2* overexpression construct at the integration site IS2.1.	This work
SbP *ADH3*-OE	TEFp-ADH3-CYC1t::IS2.1	SbP strain harboring the *ADH3* overexpression construct at the integration site IS2.1.	This work
SbP *ALD4*-OE	TEFp-ALD4-CYC1t::IS2.1	SbP strain harboring the *ALD4* overexpression construct at the integration site IS2.1.	This work
SbP *ALD5*-OE	TEFp-ALD5-CYC1t::IS2.1	SbP strain harboring the *ALD5* overexpression construct at the integration site IS2.1.	This work
SbP *ALD6*-OE	TEFp-ALD6-CYC1t::IS2.1	SbP strain harboring the *ALD6* overexpression construct at the integration site IS2.1.	This work
Sb.P *tor1*ΔΔ	*tor1*ΔΔ	SbP strain in which the *TOR1* ORF has been deleted.	This work
Sb.P *cit1*ΔΔ	*cit1*ΔΔ	SbP strain in which the *CIT1* ORF has been deleted.	This work
Sb.P *cit2*ΔΔ	*cit2*ΔΔ	SbP strain in which the *CIT2* ORF has been deleted.	This work
Sb.P *cit3*ΔΔ	*cit3*ΔΔ	SbP strain in which the *CIT3* ORF has been deleted.	This work
Sb.P *acs1*ΔΔ	*acs1*ΔΔ	SbP strain in which the *ACS1* ORF has been deleted.	This work
Sb.P *ach1*ΔΔ	*ach1*ΔΔ	SbP strain in which the *ACH1* ORF has been deleted.	This work
Sb.P *sdh1*ΔΔ	*sdh1*ΔΔ	SbP strain in which the *SDH1* ORF has been deleted.	This work
Sb.P *sdh1b*ΔΔ	*sdh1b*ΔΔ	SbP strain in which the *SDH1b* ORF has been deleted.	This work
ENT *ADH2*-OE	TEFp-ADH2-CYC1t::IS2.1	ENT strain harboring the *ADH2* overexpression construct at the integration site IS2.1.	This work
ENT *ADH3*-OE	TEFp-ADH3-CYC1t::IS2.1	ENT strain harboring the *ADH3* overexpression construct at the integration site IS2.1.	This work
ENT *ALD4*-OE	TEFp-ALD4-CYC1t::IS2.1	ENT strain harboring the *ALD4* overexpression construct at the integration site IS2.1.	This work
ENT *ALD5*-OE	TEFp-ALD5-CYC1t::IS2.1	ENT strain harboring the *ALD5* overexpression construct at the integration site IS2.1.	This work
ENT *ALD6*-OE	TEFp-ALD6-CYC1t::IS2.1	ENT strain harboring the *ALD6* overexpression construct at the integration site IS2.1.	This work
ENT *tor1*ΔΔ	*tor1*ΔΔ	ENT strain in which the *TOR1* ORF has been deleted.	This work
ENT *cit1*ΔΔ	*cit1*ΔΔ	ENT strain in which the *CIT1* ORF has been deleted.	This work
ENT *cit2*ΔΔ	*cit2*ΔΔ	ENT strain in which the *CIT2* ORF has been deleted.	This work
ENT *cit3*ΔΔ	*cit3*ΔΔ	ENT strain in which the *CIT3* ORF has been deleted.	This work
ENT *acs1*ΔΔ	*acs1*ΔΔ	ENT strain in which the *ACS1* ORF has been deleted.	This work
ENT *ach1*ΔΔ *Alias ENT1*	*ach1*ΔΔ	ENT strain in which the *ACH1* ORF has been deleted.	This work
ENT *sdh1*ΔΔ	*sdh1*ΔΔ	ENT strain in which the *SDH1* ORF has been deleted.	This work
ENT1	*ach1*ΔΔ	High acetic acid in YPD2%, does not accumulate acetic acid in YPD0.8%.	This work
ENT2	*ach1*ΔΔ TEFp-ALD4- CYC1t::IS2.1	High acetic acid in YPD2%, transient accumulation in YPD0.8%.	This work
ENT3	ACH1ΔΔ TEFp-ALD4- CYC1t::IS2.1 TEFp-ALD4- CYC1t::IS7.1	Very high acetic acid in YPD2%, permanent acetic acid accumulation in YPD0.8%	This work

^*a*^ The cassette TEFp-ALD4- CYC1t, present in the strains ENT2; ENT3 is referred to as ALD4-OE.

**TABLE 3:** Plasmids

Plasmid name	Description	Reference
pTEF-Cas9-KanMX	Plasmid containing the endonuclease Cas9. Single copy, vector backbone p414-TEF1p-Cas9-CYC1t with auxotrophic marker replaced by the genetic resistance marker KanMX.	(49) (50)
pgRNA-uni-NAT	Plasmid backbone for cloning of gRNA sequences to target specific integration sites. Multicopy, vector backbone p426-SNR52p-gRNA.CAN1.Y-SUP4t. Created by the insertion of the nourseothricin resistance marker NatMX into p426-SNR52p-gRNA.CAN1.Y-SUP4t plasmid backbone.	(49, 50)
p426hph	Backbone for construction of donor DNA. Contains the pMB1 ori (*E. coli*) and 2 micron ori (*S. cerevisiae*, multi-copy) and the hygromycin resistance marker hphMX6.	(50)
p426hph-IS2.1	p426hph backbone with homologous regions for the integration site IS7.1	(50)
p426hph-IS7.1	p426hph backbone with homologous regions for the integration site IS7.1	(50)

### Yeast culture conditions and acetic acid production assays

Overnight yeast precultures were adjusted to an OD_600_ of 0.5 (corresponding to 4 × 10^6^ CFU/mL) in 50 mL YPD (with different glucose concentrations as indicated) and incubated aerobically in a 300-mL Erlenmeyer flask equipped with a metal cap allowing passage of air. Flasks were incubated by shaking at 200 rpm and 37°C in a shaking incubator for 72 h. To obtain cell-free culture supernatants, aliquots of yeast cultures were withdrawn from the flasks and centrifuged at maximum speed (14,000 rpm) for 5 min.

### Agar well diffusion assays

Round petri dishes containing 20 mL Schaedler agar (15 g bacto agar/L Schaedler broth) were overlayed with molten soft Schaedler agar (7.5 g bacto agar/L Schaedler broth) inoculated with each one of the tested bacterial strains at a concentration of approximately 5.10^4^ cells/mL. Wells were punched into both agar layers. The resulting agar discs were carefully removed from each well with a pair of sterile thongs and discarded. Each well was then filled with 200 µL of testing solution. The testing solutions were ampicillin (100 µg/mL), pure acetic acid solutions (at 3, 6, 9, or 12 g/L in YP pH 4), and cell-free supernatant (from SbP, ENT, and ENT3 cultures). Two sets of plates were prepared, one of which was incubated inside an anaerobic jar. Plates were incubated at 37°C for 24–48 h.

### Low pH viability assays

Overnight cultures were harvested by centrifugation at 3,000 rpm for 5 min, washed with distilled water once, and incubated at OD_600nm_ = 1 and 37°C for 3 h in (i) a simulated gastric environment comprising an aqueous solution containing 5 g/L NaCl, pH 1.7, and (ii) a control saline solution containing 5 g/L NaCl, without pH adjustment (pH 6.8).

Viability was determined by colony counting on nutrient agar plates of cells that had been exposed for 3 h to acidic saline solution (NaCl 0.5%, pH1.7) compared with 3 h exposure to saline at pH 6.8. In this way, only the effect of low pH is taken into consideration and any possible osmotic effect is excluded.

### CRISPR/Cas9-mediated gene deletion and overexpression

#### Deletion experiments

Two gRNAs were used targeting each gene. The first gRNA targets within the first nucleotides in the open-reading frame (ORF), and the second gRNA targets within the last ones. An 80-bp oligomer is given as repair template, consisting of 40 bp immediately upstream of the first cutting site and 40 bp immediately downstream of the second one. As a result, the ORF is removed and no exogenous DNA is inserted.

#### Overexpression experiments

Each overexpression target gene has its original sequence amplified from SbP and cloned under the strong and constitutively expressed TEF1 promoter and the CYC1 terminator.

Modification of each gene was tested in the SbP and ENT strains. All tested genes were individually inserted in the same previously tested neutral site, named IS2.1 (50).

For construction of the ENT3 strain, ALD4-OE copies were inserted at the sites IS2.1 and IS7.1 (50).

### Animals

The mice (C57/Bl6 JAX) were obtained from the internal stock deviated from Charles River. Before experiments were initiated, the mice were acclimatized for at least 2 weeks in which they were handled to reduce stress-related bias in the experiments. Prior to the experiment, the mice were randomized over the treatments by balancing for body weight.

### Yeast survival through mice GI tract

Yeasts were grown overnight in YPD at 30°C. Aliquots containing a yeast suspension of 5 × 10^9^ CFU/mL were prepared in PBS with 30% glycerol and stored at −80°C until use. A yeast suspension (10^9^ CFU in 200 µL) or control medium (PBS + 30% glycerol) was administered to 8-week-old healthy mice (each treatment *n* = 5) by single-dose intragastric gavage.

Feces were collected before gavage (0 h) and at 2 h, 4 h, 6 h, 8 h, 10 h, and 24 h and then once a day from day 2 to day 7. Feces were weighed and suspended in PBS/glycerol using 3 µL PBS/mg of feces as described by Sovran et al. (51). Samples were immediately stored at −80°C until plating. Appropriate dilutions were plated onto modified YPD agar (0.5% yeast extract, 1% peptone, 1.5% agar, and 2% glucose) containing 100 µg/mL ampicillin and incubated at 30°C for 2 days.

## SUPPLEMENTAL MATERIAL

Supplemental figures and tables aem.00325-24-s0001.pdf Figures S1 to S5 and Tables S1 to S3.

ASM does not own the copyrights to Supplemental Material that may be linked to, or accessed through, an article. The authors have granted ASM a non-exclusive, world-wide license to publish the Supplemental Material files. Please contact the corresponding author directly for reuse.
