# Supplementary material for: Enhancing probiotic impact: engineering Saccharomyces boulardii for optimal acetic acid production and gastric passage tolerance
Source: Appl Environ Microbiol. 2024 May 16;90(6):e00325-24. doi: 10.1128/aem.00325-24 (PMC11218656; doi:10.1128/aem.00325-24)
Supplement: Supplemental figures and tables — Figures S1 to S5 and Tables S1 to S3. [file aem.00325-24-s0001.pdf]

Supplementary materials

Supplementary Figures

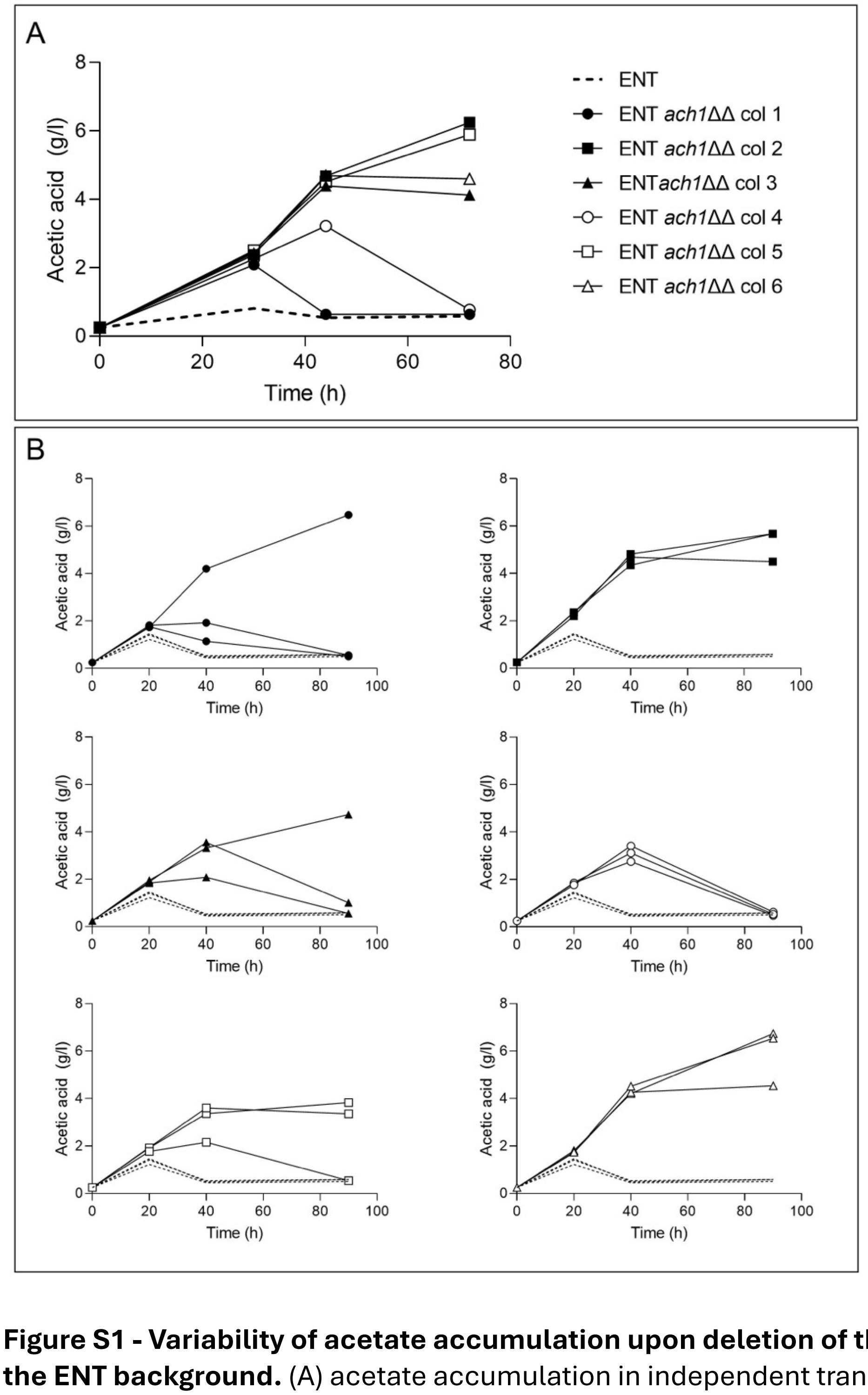

Figure S1 - Variability of acetate accumulation upon deletion of the ACH1 gene in the ENT background. (A) acetate accumulation in independent transformants. (B) Repetition of individual transformants. The ENT wild type is represented by dashed lines and transformants by full lines, (●) colony 1, (■) colony 2, (▲) colony 3, (○) colony 4, (□) colony 5 and (△) colony 6. Cells were propagated in YPD2% at 37°C, 200 rpm for 72 h.

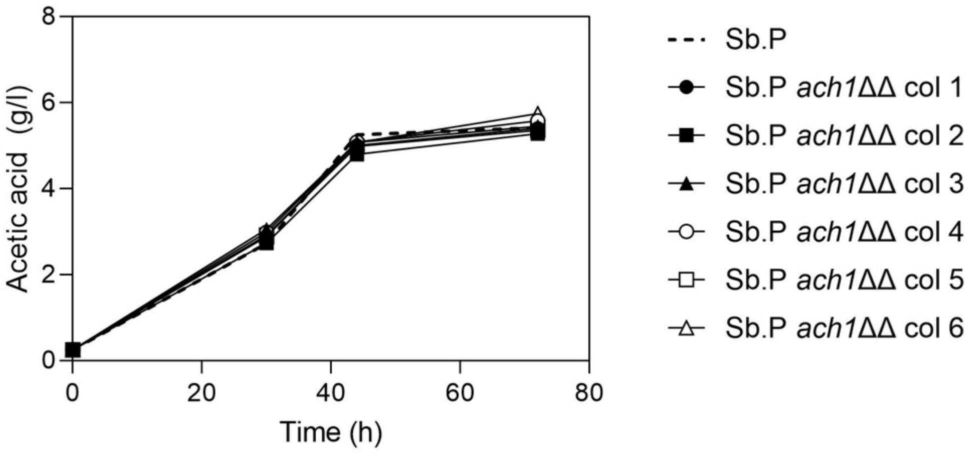

Figure S2 - Acetate accumulation upon deletion of the ACH1 gene in the SbP background. Acetate accumulation displayed by independent transformants. The SbP wild type is represented by dashed lines and transformants by full lines, (●) colony 1, (■) colony 2, (▲) colony 3, (○) colony 4, (□) colony 5 and (△) colony 6. Cells were propagated in YPD2% at 37°C, 200 rpm for 72 h.

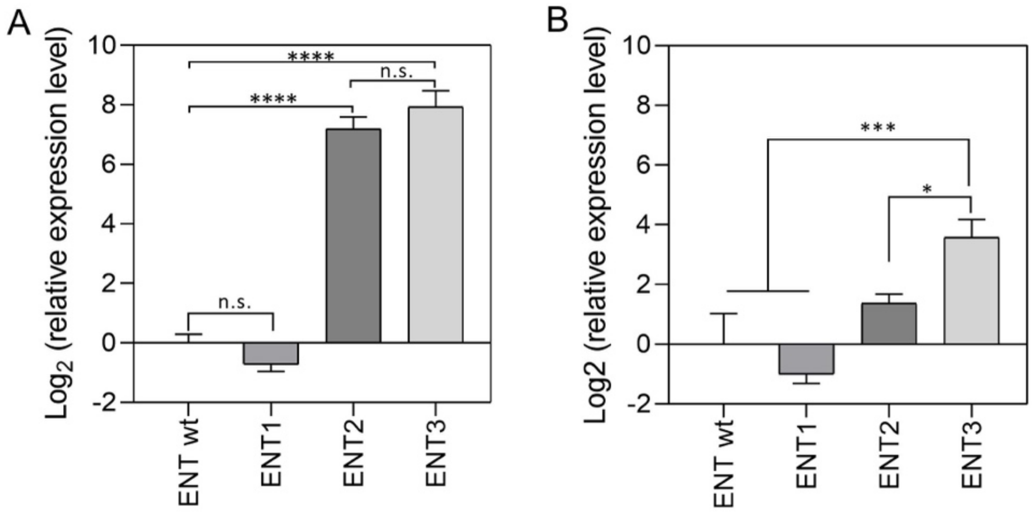

Figure S3 – Expression levels of ALD4 at 8h (A) and 24h (B) of growth in the ENT, ENT1, ENT2 and ENT3 strains. Strains were grown in YPD2% at 37°C. The comparative Ct analysis was performed with qBase+ (Biogazelle). ACT1, 18s, SCR1 were selected as stable reference genes for samples harvested at 8h, and 18s, SCR1 for samples harvested at 24h. Normalized relative quantities (NRQ) were scaled to the parent ENT strain. Statistical analysis was done using Ordinary One-way ANOVA (Tukey's multiple comparisons test, *$p \leq 0.05$, **$p \leq 0.01$, ***$p \leq 0.001$, ****$p \leq 0.0001$, ns – non-significant). Data represent mean values ± SD from three independent colonies.

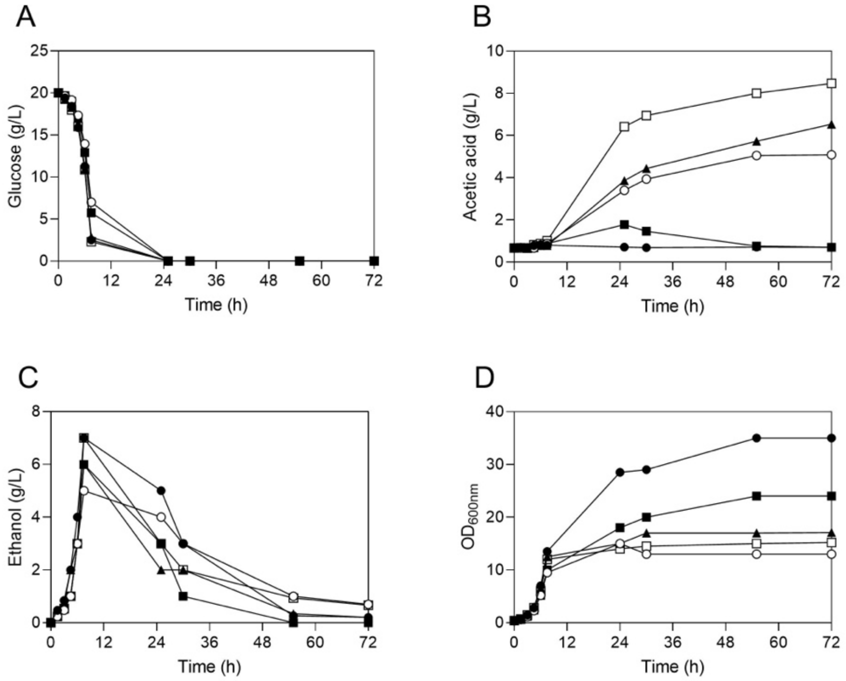

Figure S4. ***S. cerevisiae*** **and** ***S. boulardii*** **fermentation in YPD2% at 37°C**. (●) S. cerevisiae S288c, (○) *S. boulardii* SbP, (■) *S. boulardii* ENT, (▲) *S. boulardii* ENT1 and (□) *S. boulardii* ENT3. (A) glucose consumption, (B) acetic acid accumulation. (C) ethanol concentration and (D) cell growth as a function of time.

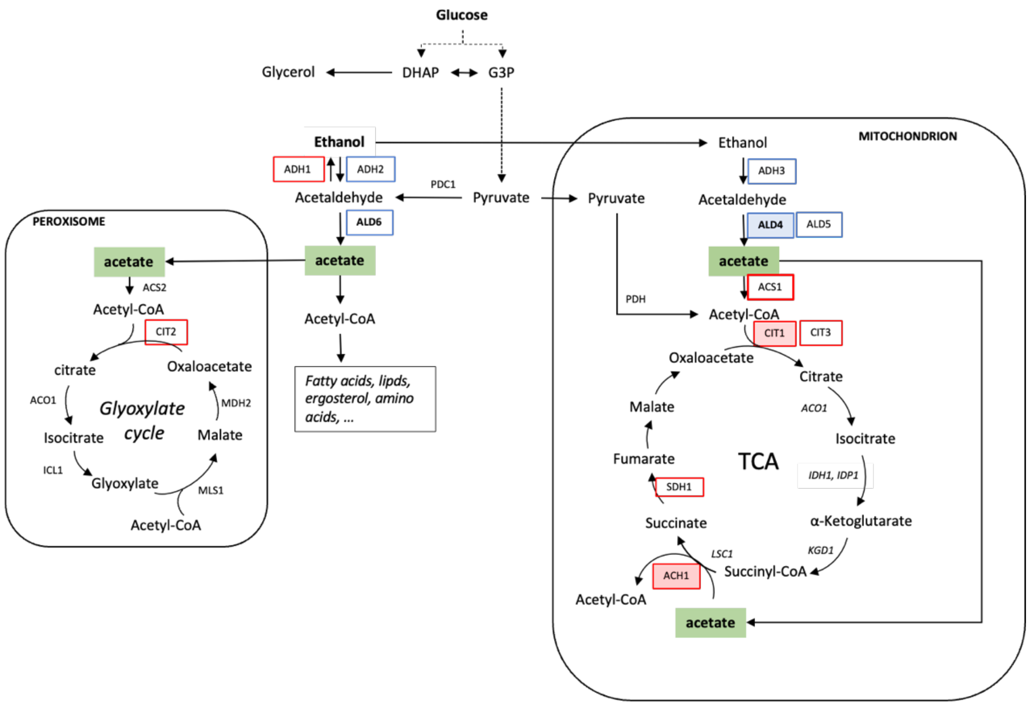

Figure S5. Acetic acid production and consumption pathways in yeast and the targets for genetic engineering. Deletion targets are marked in red boxes. Overexpression targets in blue boxes. Adapted from (1, 2).

Supplementary Tables

Supplementary Table 1

Supernatant pH and acetic acid concentrations after propagation in YPD2%, 37°C, 72h

**Strain**	**AA (g/L) at 72h in YPD2%**	**Supernatant pH**
SbP	5.0	4.07
SbP SDH1^{sc} (no acetate)	0.25	4.89
ENT	0.20	4.69
ENT3	8.50	3.73

Average supernatant pH of *S. cerevisiae* strains is 5.3 (3)

Average supernatant pH shown by different *S. boulardii* strains

**Strain**	**Average supernatant pH**
S. cerevisiae	5.30 (3)
S. boulardii	4.89
S. boulardii ach1Δ	4.08
S. boulardii ach1Δ ALD4-OE (2x)	3.93
S. boulardii ach1Δ ALD4-OE (4x)	3.78

Supplementary Table 2

List of gRNA target sequences

**Gene/Region**	**gRNA1 target sequence**	**gRNA2 target sequence**
*Overexpression**		
IS2.1	ATCAACCACAGTGAACGCCG	-
IS7.1	TAGCATCGTGCATGGGATAG	-
*Deletion***		
TOR1	AACCGCATGAGGAGCAGATT	GAATGGGCACCATCCAATAT
CIT1	GCCCTATCGATGATTAATTG	CAAGACAATGTCAAAATATG
CIT2	ATAGGACTTTGGCCTTTCAA	CATCCATGCTCAAGATGTAA
CIT3	ATGGTACAAAGGCTTCTACC	GACCCTCCAAGTTCAAACTC
ACS1	TTAGATGTCTAACAATGCCA	TCTGCCGTACAATCATCAAA
ACH1	ATTTGTTAAAGCAGAGAGTT	AGCTGTCGACCTTCATTGTA
SDH1	AATTAGTAGGCTCTTACAGT	AACGCAGGGCTCTGTAAACG
SDH1b	CGACCATGAATATGATTGTG	GTACTTGATTTTTACAGGCG

**The overexpression cassettes of each tested gene were integrated at the region IS2.1. For construction of the ENT3 strain, the ALD4-OE cassette was inserted at the sites IS2.1 and IS7.1.*
*** Two gRNAs were used targeting each gene. The first gRNA targets within the first nucleotides in the open reading frame (ORF) and the second gRNA targets within the last ones.*

Supplementary Table 3

List of donor DNAs

Gene/Region	Sequence
TOR1 repair Fw	GTAAAGTGAAACATACATCAACCGGCTAGCAGGTTTGCATTGATCTGCGGTGT CATTTTTCATTTCGTGCTTTGTTTACTATTTATTT
TOR1 repair Rv	AAATAAATAGTAAACAAAGCACGAAATGAAAAATGACACCGCAGATCAATGC AAACCTGCTAGCCGGTTGATGTATGTTTCACTTTAC
CIT1 repair Fw	CGTGTTTGAATAGTCGCATACCCTGAATCAAAAATCAAATTTTCCCTTCGTAAA TAGTATTATATTGCTATATGTTTTGCCTTATTTT
CIT1 repair Rv	AGAAAATAAGGCAAAACATATAGCAATATAATACTATTTACGAAGGGAAAAT TTGATTTTTGATTCAGGGTATGCGACTATTCAAACA
CIT1 repair Fw	CGTGTTTGAATAGTCGCATACCCTGAATCAAAAATCAAATTTTCCCTTCGTAAA TAGTATTATATTGCTATATGTTTTGCCTTATTTT
CIT2 repair Fw	AAAGAAAAATATGCAGAGGGGTGTAAAAGTAGGATGTAATCCAATTTTCTTGT TACTAGTATTATTAAAACAAAAAGTTTTGAGAACC
CIT2 repair Rv	GGTTCTCAAAACTTTTTGTTTTAATAATACTAGTAACAAGAAAATTGGATTACA TCCTACTTTTACACCCCTCTGCATATTTTTCTTT
CIT3 repair Fw	ATCAAGAATTTATACATAGACGCCGCTAAATAATTGAATACAAACGCAGTTCC AATTTACAAGAATGCTTCGTTTGCTATTACAATAT
CIT3 repair Rv	ATATTGTAATAGCAAACGAAGCATTCTTGTAAATTGGAACTGCGTTTGTATTC AATTATTTAGCGGCGTCTATGTATAAATTCTTGAT
ACS1 repair Fw	CGAAAAAAAAAAAGTCGTCAATATAAAAAGGAAAGAAATCATCAAACACAGT GGGGCAATGTCTTTCTAGTAGTTTTGATATGTTTGG
ACS1 repair Rv	ACCAAACATATCAAAACTACTAGAAAGACATTGCCCCACTGTGTTTGATGATT TCTTTCCTTTTTATATTGACGACTTTTTTTTTTTC
ACH1 repair Fw	CAAACAACACATTTCTTTTTTTCTTTTTCACATATTGCACTAAATGTTTGTGCGC AAACCAAGAGATGAGTATTTAACAAAAAAAAGA
ACH1 repair Rv	TCTTTTTTTTGTTAAATACTCATCTCTTGGTTTGCGCACAAACATTTAGTGCAAT ATGTGAAAAAGAAAAAAAGAAATGTGTTGTTTG
SDH1 repair Fw	GAAGAGTATGATATTCTTTTCCGCAAAATACAATGAGGTTCAAACTTTATTCTC GTTCTTCTTCGTACTATGAAATTGGATTTTTTTC
SDH1 repair Rv	GAAAAAAATCCAATTTCATAGTACGAAGAAGAACGAGAATAAAGTTTGAACC TCATTGTATTTTGCGGAAAAGAATATCATACTCTTC
SDH1b repair Fw	AATACGTATATCTATATACATGTATACACGTGAGCTAATAAATTTTCTTATTTA TTTATTTATTTATTTTGGAGGGCAAACTTATTTA
SDH1b repair Rv	TAAATAAGTTTGCCCTCCAAAATAAATAAATAAATAAATAAGAAAATTTATTA GCTCACGTGTATACATGTATATAGATATACGTATT

References

1. Lian J, Si T, Nair NU, Zhao H. 2014. Design and construction of acetyl-CoA overproducing Saccharomyces cerevisiae strains. Metab Eng 24:139-49.
2. Orlandi I, Coppola DP, Vai M. 2014. Rewiring yeast acetate metabolism through MPC1 loss of function leads to mitochondrial damage and decreases chronological lifespan. Microb Cell 1:393-405.
3. Offei B, Vandecruys P, De Graeve S, Foulquie-Moreno MR, Thevelein JM. 2019. Unique genetic basis of the distinct antibiotic potency of high acetic acid production in the probiotic yeast *Saccharomyces cerevisiae var. boulardii*. Genome Res 29:1478-1494.
